# First Case of an Infant with COVID-19 in the Middle East

**DOI:** 10.7759/cureus.7520

**Published:** 2020-04-03

**Authors:** Amani Mansour, Rola Atoui, Kamal Kanso, Rami Mohsen, Youssef Fares, Jawad Fares

**Affiliations:** 1 Pediatrics and Adolescent Medicine, Lebanese University Faculty of Medicine, Beirut, LBN; 2 Infectious Diseases, Lebanese University Faculty of Medicine, Beirut, LBN; 3 Neuroscience Research Center, Lebanese University, Beirut, LBN; 4 Neurological Surgery, Northwestern University Feinberg School of Medicine, Chicago, USA

**Keywords:** covid-19, coronavirus, sars-cov-2, pediatrics, infants, pandemic, lebanon, middle east, gastrointestinal symptoms, children

## Abstract

The novel coronavirus (COVID-19) has been declared a worldwide pandemic. It was initially thought to spare children and adolescents as significantly smaller number of cases have been reported in the pediatric population in comparison to adults. Here, we report the case of a 16-month-old female infant from Lebanon who presented with fever and severe diarrhea and tested positive for COVID-19. Her symptoms started six days prior to presentation with no cough, rhinorrhea, or other respiratory manifestations reported. Chest radiography showed lobar consolidation and bronchial infiltrates. Blood culture was positive for *Streptococcus pneumoniae*. Stool and urine cultures were negative. She was treated with ceftriaxone and metronidazole. Her RT-PCR test was negative after five days of treatment, suggesting that children can clear the virus faster than adults. The patient likely contracted the virus from her parents, who because of the fear of social stigma hide recent history of respiratory illness. These findings serve as a practical reference for the clinical diagnosis and medical treatment of children with COVID-19.

## Introduction

The novel coronavirus disease 2019 (COVID-19) is the result of an infection with the severe acute respiratory syndrome coronavirus 2 (SARS-CoV-2), a member of *Betacoronavirus*. Initial cases were detected in Wuhan, a city in China, near the end of 2019 [1]. The virus is believed to replicate in the respiratory system during the prodromal period, and exhibits human-to-human transmission patterns through respiratory droplets and contact routes [1,2]. The disease causes mild to severe illness, with symptoms appearing 2-14 days after exposure and most commonly comprising fever, cough, and shortness of breath. Most severe illness occurs in older adults but comparison with the pediatric population can be challenging as documented cases in infants and children have been scarce [3,4].

As of mid-March 2020, COVID-19 has spread rapidly and widely to become a global pandemic [5]. Outbreaks have been reported in China, South Korea, Iran, Italy, Spain, and the United States. Many other European, Asian, African, and North and South American countries have been reporting cases [6]. Nevertheless, the Middle East countries have been lagging in terms of publishing clinical outcomes [7].

## Case presentation

Here, we report the first case in the Middle East of a 16-month-old Lebanese female, previously healthy, who had symptomatic COVID-19.

The patient was transferred from another hospital due to increasing hypoactivity and severe diarrhea. The referring hospital had ruled out coronavirus infection due to the absence of cough. Parents reported that the infant was healthy and had regular food intake until symptoms started six days prior to presentation. They denied exposure to or contact with infectious risk factors and affirmed that no cough/rhinorrhea symptoms were present.

Upon examination, the patient was febrile (40°C) with a respiratory rate of 24 breaths per minute and a heart rate of 166 bpm. Chest auscultation revealed rhonchi. Differential diagnoses included gastroenteritis, pneumoniae, and COVID-19, and the patient was put on isolation as a preliminary measure.

Laboratory studies revealed leukocytosis with a white cell count of 15,000 cu mm (range: 3,400-9,600) and elevated C-reactive protein level reaching 231.16 mg/L (range: 0-5) (Table 1).

**Table 1:** Laboratory test results of the infant upon presentation. BUN: blood urea nitrogen; EGFR: estimated glomerular filtration rate; SGPT: serum glutamic pyruvic transaminase; CRP: C-reactive protein; RBC: red blood cell; MCV: mean corpuscular volume; MCH: mean corpuscular hemoglobin; RDW: red cell distribution width; MPV: mean platelet volume; PDW: platelet distribution width; ANC: absolute neutrophil count; ALC: absolute lymphocyte count. *Values not within normal range.

Laboratory Tests	At Presentation	Normal Range
Chemistry		
Phosphorus	3.15 mg/dL	>18 years: 2.7-4.8
Na^+^	137.00 mmol/L	136.00-145.00
K^+^	3.93 mmol/L	3.50-5.10
CL^-^	104.50 mmol/L	98.00-107.00
CO_2_	*16.40 mmol/L	23.00-29.00
Creatinine	*0.21mg/dL	0.51-0.95
BUN	*4.00 mg/dL	6.00-20.00
Calcium	9.33 mg/dL	8.50-10.50
Magnesium	2.15 mg/dL	1.70-2.60
EGFR	210.33	>60.00 mL/min/1.72 m^2^
SGPT	21.00 U/L	5.00-41.00
Bilirubin total	0.60 mg/dL	0.20-1.00
Bilirubin direct	*0.24 mg/dL	0.00-0.20
Serology		
CRP	*231.16 mg/L	0.00-5.00
Hematology		
RBC	*3.29 x10^6^ cu mm	3.92-5.13
Hemoglobin (Hb)	*8.40 g/dL	11.50-16.00
Hematocrit (Ht)	*24.80%	35.50-44.90
MCV	*75.00 f L	78.00-98.00
MCH	*25.50 pg	27.00-31.00
RDW	14.60%	12.00-16.00
Platelets	*524000 cu mm	150000-375000
MPV	8.20 um^3^	6.00-11.00
PDW	12.80 %	11.00-18.00
WBC	*15,500 cu mm	3400-9600
Neutrophils	*74.40%	40.00-65.00
ANC	*11.50 cu mm	1.56-6.45
Lymphocytes	*15.80%	25.00-40.00
ALC	2.45 cu mm	0.95-3.07
Monocytes	*9.30%	2.00-8.00
Monocyte count	*1.44 cu mm	0.26-0.81
Eosinophils	0.50%	0.00-4.00
Eosinophil count	0.08 cu mm	0.03-0.48
Basophils	0.00%	0.00-1.00

**Figure 1:**
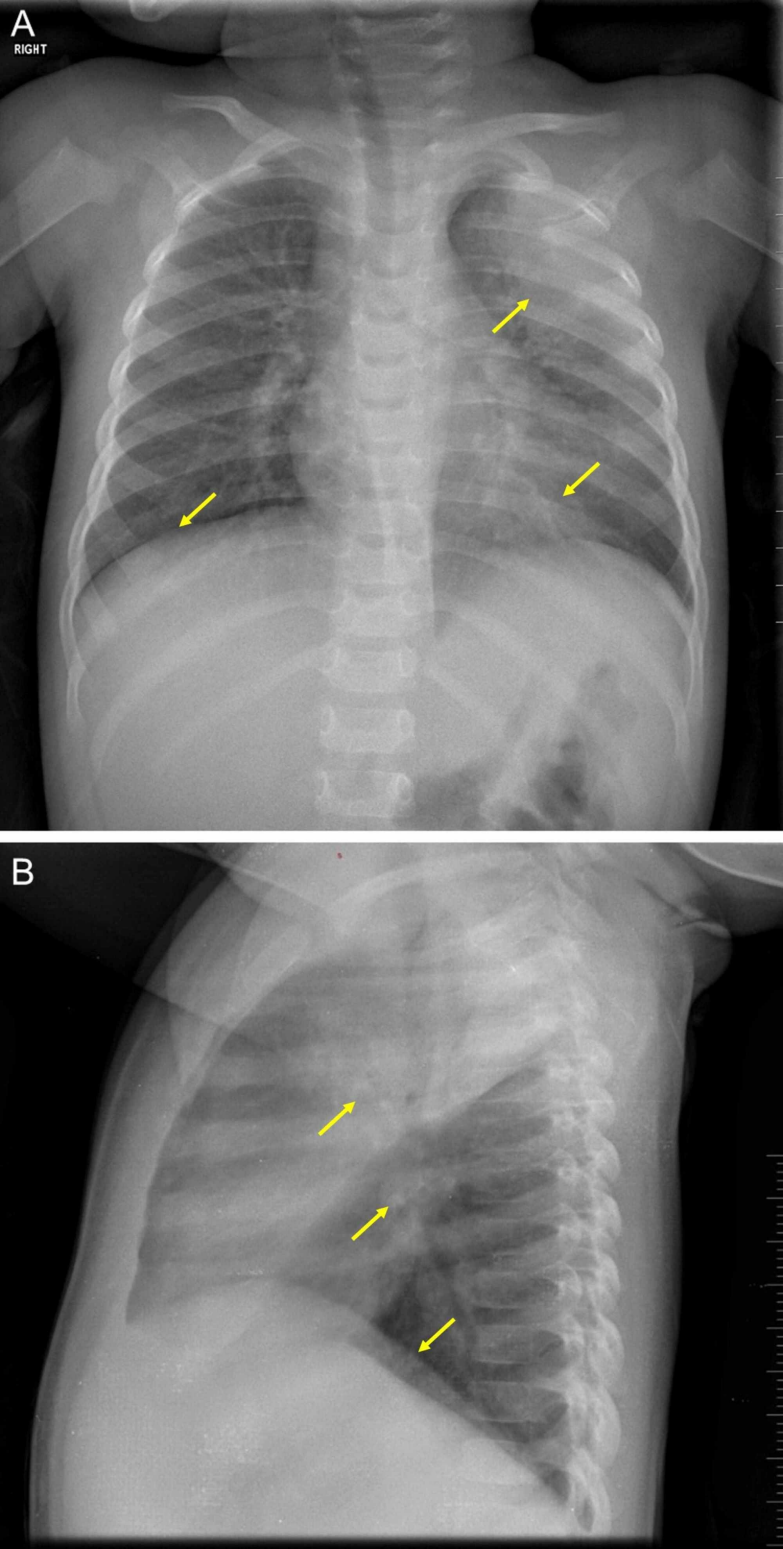
A chest radiograph. Imaging of the infant’s chest shows large consolidation at the left apical lobe with bronchial infiltrates that are dominant at the left base, and asymmetrical lung bases (A). The heart size is normal, the rib cage is intact, and the diaphragmatic arches are in normal position (A). A discrete blunting at the left pleural sinus can be observed (B).

Cultures for blood, urine, and stool were taken. A chest radiograph showed left upper lobe consolidation and bilateral lower lobe infiltrates (Figure 1). This warranted nasopharyngeal swabs for an RT-PCR to test for SARS-CoV-2. 

In the meantime, the patient was put on a hydration regimen and was started on ceftriaxone (75 mg/kg/day) and metronidazole (10 mg/kg, every eight hours). On day 2 of admission, the patient became afebrile and exhibited improvement in physical activity. On day 3, the result of the RT-PCR test returned positive for COVID-19. Blood culture was positive for *Streptococcus pneumoniae*. Stool and urine cultures were negative.

Upon further investigation, the father admitted that he had ‘flu-like’ symptoms two weeks before presentation but denied travel history or contact with a defined case of COVID-19. The mother also confessed having similar symptoms but did not seek medical consultation at the time. This hinted that the patient likely contracted the virus from her parents, who, in turn, might have been infected through community transmission. The family was transferred to a designated quarantine for isolation. On day 5, the RT-PCR test of the infant was negative, and the patient’s symptoms had resolved.

## Discussion

Cases of COVID-19 in children are not as rare as they might have been thought [8,9]. This is the first case reported from the Middle East that involves a 16-month-old female infant. Around 2.4% of cases with COVID-19 were reported to be among the pediatric population in China [10]. Ages ranged between 3 and 14 years, with males being predominantly affected [10-12].

Uniquely, our patient presented with fever and diarrhea; cough and other respiratory symptoms were not reported. Previous COVID-19 studies in the pediatric population noted that common symptoms include fever, cough, sore throat, and rhinorrhea. Diarrhea has not been reported yet [6,10]. This warrants a more comprehensive definition of the symptoms that govern COVID-19 in the pediatric population, as gastrointestinal symptoms have been documented in our case and among adults [13].

The RT-PCR test was negative after five days of treatment and 11 days after the onset of symptoms. This suggests that children might clear the virus more rapidly than adults. Similarly, previous research in children indicates that the RT-PCR test becomes negative within 12 days (range: 6-22) after the presentation of symptoms [6]. The first RT-PCR test took two days for the result to generate, whereas the subsequent test was much faster, and the result was obtained on the same day due to the expansion of the testing capacity by the Lebanese Ministry of Public Health.

Fear of social stigma drove the patient’s parents to hide information of their respiratory tract illnesses. It is important to address this issue at public health levels and to stress and highlight the social responsibility associated with reporting any relevant medical data related to the COVID-19 pandemic. Moreover, health communication and promotion strategies must be improved to increase awareness and literacy on the current pandemic, and brush off rumors and misinformation that can cause fear and panic [14].

## Conclusions

This is the first case reported from the Middle East on an infant presenting with fever and diarrhea that tested positive for COVID-19. Cases of COVID-19 in children are not as rare as they might have been thought. COVID-19 related pediatric symptoms can exhibit an array of presentations, including diarrhea. Most cases recover well with symptomatic care. This case serves as a practical reference for the clinical diagnosis and medical treatment of infants with COVID-19.
